# The Annual Statistical Return

**Published:** 1918-11-16

**Authors:** 


### KING EDWARD'S HOSPITAL FUND FOR LONDON.

### The Annual Statistical Return.

The Statistical Report * for 1917 on the ordinary expenditure of the hospitals of London deals with 108 institutions, as against 109 in 1916. The number of beds dealt with—namely, 11,729—resembles so closely the 11,406 of the preceding return that from figures representing expenditure, numbers of patients, and the like fairly satisfactory conclusions may be drawn by the comparison of the two years. Mr. Maynard shows in his interesting and conveniently arranged compilation that the total number of in-patients admitted during 1917 was 147,598, while 1,152,022 patients attended at the out-patient departments. The figures for 1916 were 153,512 and 1,251,015 respectively.

Naval and military patients treated by understanding with the authorities have slightly increased in number, from 27,328 to 28,013, 31 per cent. of the beds in both years being used for this purpose. The beds in London hospitals have during the war been increased by 1,997, but the beds available for civilian patients have been reduced by 1,659.

The total ordinary expenditure of the 108 hospitals, with that of St. Bartholomew's and the Cancer Hospital added, amounted to £1,719,030, as against £1,513,834. The working expenditure has increased during the war, *i.e.*, since the year 1913, by £311,540, or by 27.36 per cent. In the preliminary statement signed by the honorary secretaries it is argued that as a large sum of money (£268,980 in 1917) was paid by naval and military authorities the expenses to be met by the hospitals should be calculated as having been reduced by that amount; in other words, that only a smaller amount, £1,450,070, came out of the normal sources of hospital revenue. This to some may be thought a confused way of presenting simple figures, mingling as it does income with expenditure. In any case paying patients have been a feature of hospital work we are glad to note. It is a change which the public will welcome, and much more attention would have been given to it had there been no war. Revenue from paying patients is therefore quite a normal thing; it is abnormal not in character, in respect of the naval and military cases, but in extent. Nobody can confidently say, moreover, that the relatively small sum of £268,000 in respect of paying patients in 1917 would have been abnormal had not the paying-bed movement been interrupted by the war.

In presenting the figures as the honorary secretaries do there is a suggestion that the payments made for sailors and soldiers approximately meet the outgoings on their account. This is not so. The 4s. 9d. per head paid since October 1, 1917, and the 4s. before that date, in very few hospitals is equal to the cost per occupied bed.

The Statistical Report is presented in the customary manner, the hospitals being arranged in groups and the cost computed under main headings. As between the groups the larger hospitals with medical schools again show a greater average cost per bed than those without schools—namely, £123, as compared with £92. Taking the general hospitals individually this result does not always hold good; for instance, the cost at University College Hospital, which has a medical school, was £77, while that at the Great Northern Central, without a school, was £100.

**In examining the comparisons contained in these groups of hospitals we have been again struck by a condition of preparing the accounts, which has been pointed out more than once in these columns. This is in respect of the allocation of cost as between out-patients and in-patients. In one group the proportion of the expenditure reckoned as having been incurred by out-patients varies as greatly as 15 per cent., although the out-patient work varied very slightly. The allocation of expense is left very much to the individual hospital secretary or accountant, and this fact must largely vitiate the value of the figures. No result at all valuable for purposes of accurate comparison can be attained until this system is amended either by the appointment of a general overseeing auditor or by a reversion to the old custom of a flat rate for out-patients for each group of hospitals.**

* Published for King Edward's Hospital Fund for London by Spottiswoode, Ballantyne and Co., Ltd., 1 New Street Square, E.C. 4. Price 1s. net, 1s. 2d. post free.

